# Round the Hospitals

**Published:** 1919-08-09

**Authors:** 


### ROUND THE HOSPITALS.

The King's well-chosen words in receiving addresses of congratulation from the London County Council and from English Presbyterian and Protestant dissenting ministers will find an echo in the hearts of many nursing sisters, taking holiday may be before the commencement of heavier tasks. "Freedom has been won for the nations of the world, but at a price of incalculable suffering. It rests with us to show ourselves worthy of the sacrifices others have made and to build up a new world to replace the shattered fabric of the old." Many of our readers whose privilege it is to care for the weak and helpless, to protect infant life, and extend their aid to the infirm in body and mind, know full well from personal experience how truly the King spoke when

**ROUND THE HOSPITALS—(*continued*).**

he declared that these are matters very near to the hearts of the Queen and himself. We are greatly blessed in this country in having the active and cordial association of the Royal family in the work which lies ahead. There could be no more powerful auxiliary in combating the great enemy to all social reforms, public indifference.

Notice is given that a petition has been presented to His Majesty in Council by the British Red Cross Society praying for the grant of a supplemental Charter. The King has referred the petition to a Committee of the Lords of Council, and petitions for or against such grant must be delivered at the Privy Council Office on or before August 16. The matter is one of considerable interest to nurses. By the terms of its Charter the sphere of the British Red Cross Society is limited to the supply of aid to the sick and wounded in time of war. To enable it to lead the way in the relief of sickness and distress and in preventive work now that war is at an end it is necessary for the Society to obtain fresh powers, and until this is done this mighty engine must let its wheels run slack. The International Red Cross delegates who have been holding meetings at Geneva have now come to an understanding as to the future developments which may legitimately be aimed at, and once the new Charter is granted an enormous extension of voluntary aid may be anticipated. It was a great safeguard for professional nurses that some of the acknowledged leaders of their profession were present at the Geneva conferences.

There are several admirable organisations ready to extend advice and financial aid to nursing sisters who are in difficulties. There is the Junius Morgan Fund for helping the many thousand members of the Royal National Pension Fund for Nurses. There is the College of Nursing ready to extend expert legal advice (sometimes the best aid of all) to any of its 14,000 members. There is the Nation's Fund for Nurses ready to help any nurse who is ill or disabled, and there are the Edith Cavell Homes of Rest with facilities for granting free holidays and long periods of rest to those in need of them. And there are other organisations which undertake similar work. Yet we constantly see references in the press to the misfortunes of Army Sisters who are left without a helping hand in their invalided state after the long stress of the war years. There has always been a great lack of co-ordination in the nursing services, and so far as Army sisters are concerned the chief need was for a Bureau of Aid capable of dealing sympathetically and exhaustively with each case on its own merits.

It is very good news to hear that this need has been met in the best possible manner. Sir Worthington Evans, the Pensions Minister, has announced that Queen Alexandra has graciously consented to be the President of the Pensions Nursing Service, which has been provided as a necessary adjunct to the establishment of a medical services division of the Ministry. The medical services are to be furnished with hospitals of their own, and two have already been taken over. The British Red Cross Society is lending its aid, and there are to be convalescent centres in different parts of the kingdom. We learn with pleasure that Miss M. E. Davies, late matron of the King George Hospital, Stamford Street, S.E., has been appointed Matron-in-Chief of the Pensions Nursing Service.

We learn with much satisfaction that as the result of the garden party given by gracious permission of His Majesty at St. James's Palace last month the sum of £1,500 has been realised and the total paid into the coffers of the Nation's Fund for Nurses. The fête took place in inclement weather, and its success speaks much for the zeal with which it was supported.

At the Investiture held at Buckingham Palace on July 31 the King personally conferred the decoration of the Royal Red Cross, First Class, on Sisters Patricia O'Curran, Edith Sutton, and Louise Thurling, Q.A.I.M.N.S.R.; on Sisters Mollie Jones, Gertrude Lulham, Nellie Nicholls, and Edith Parsloe, T.F.N.S. Royal Red Cross, Second Class, on Sisters Susan Baxter, Edith Carter, Gertrude Custance, Mary Colston, Sybil Gardiner, Joan Heinig, Lilian Jeans, Ethel Webb-Johnson, Agnes Kinnear, Catherine Lewis, Henrietta Mackay, Anne Mathieson, Dorothea Rudman, and Elizabeth Spensley, Q.A.I.M.N.S.R.; on Sisters Amelia Derry, Eliza Harrison, and Staff Nurse Florence McKellar, T.F.N.S.; on Sister Millicent Rutherford-Harris, B.R.C.S.; on Sister Agnes Bonnar, Australian A.N.S.; on Mrs. Ruth Cowan; the Countess of Dudley and Miss Norah Webb-Johnson, V.A.D. The King conferred the Military Medal on Sister Jane Spence and Staff Nurse Ethel Garrett, Q.A.I.M.N.S.R., and on Sister Julia Herbert, T.F.N.S. All these ladies were subsequently received by Queen Alexandra at Marlborough House.

At the Investiture held on August 2 at Buckingham Palace, the King personally conferred decorations on members of the nursing services: Royal Red Cross, First Class, Matron Constance Grasett, Civil N.S.; Royal Red Cross, Second Class, Sister Eliza Watt, Q.A.I.M.N.S., India; Matron Madeline Harrower and Staff Nurse Florence Runton, Q.A.I.M.N.S.R.; Matron Eleanor Mason and Miss Margaret Edwards, Civil N.S.; Miss Mary Molloy and Miss Lilian Trotter, B.R.C.S.; Miss May Keyser, V.A.D. These ladies were subsequently received at Marlborough House by Queen Alexandra.

The War Medal ribbon is now ready and the very precious two inches to which all ranks are entitled who are eligible for the War Medal will be issued to applicants. It is striped in orange, white, and black, with a border of royal blue. It is estimated that 8,000,000 persons are eligible for this decora-

**ROUND THE HOSPITALS**—(*continued*).

tion, which even at two inches only a head is estimated (we have not verified the figures) to run into 250 miles of ribbon. Demobilised members of Q.A.I.M.N.S.R. should apply for it to the Secretary, War Office, (A.M.O.4), Adastral House, E.C.4; demobilised members of the T.F.N.S. to the Secretary, War Office, (T.V.4), 80 Pall Mall, S.W.1 A little patience is indicated.

---

ACCORDING to the terms of the Peace Treaty, a permanent organisation is to be formed to promote international adjustment of labour, and an international conference is to be held every year in furtherance of its objects. The first Conference is to be held in Washington next October to discuss the 8-hour day, or 48-hour week, the employment of women before and after childbirth, and other subjects. It is obvious that nurses' interests will be closely affected by these deliberations, and the Council of the College of Nursing was recently asked to send a representative to take part in a deputation to Mr. Barnes requesting the direct representation of women at Washington in this conference. It was agreed that the men delegates should have women advisers on all matters affecting the welfare of women.

---

CONSIDERABLE interest attaches to the test questions set for the sister tutor studentships at King's College for Women, reported in our last issue. They are commendably free from the spirit which encourages cram, and the professional subjects on which the candidates were invited to declare their knowledge were particularly well chosen. It goes without saying that the questions on general knowledge were merely feelers whereby indications could be gained of the students' bent, and had no decisive bearing on the award. But candidates will always be found to pore over such questions set in previous years with an eye to discovering what kind of things they are expected to know, and we cannot but deem it unfortunate that the unhappy examinees were called on to devote part of their time to giving an account of "any well-known religious controversy." As wisely might an examiner try to test a student's knowledge of art by asking for particulars of persons who have succeeded in painting with their toes. If by ill-luck we have the history of any religious controversy at our finger-ends, the best thing to do with it is to cast it on the dung-hill of oblivion.

---

THE Nightingale Fund offers three scholarships, tenable for one year at King's College Hospital for Women, to nurses trained in the Nightingale School. The successful candidates at the recent examination were Miss Gladys Verena Hillyers, Miss Theodora Mamie West Watson, and Miss Mary Elizabeth Gordon Milne. We feel sure that as time goes on the managers of the leading nurse training-schools will realise that the best class of probationer is attracted far more by the higher opportunities of study and self-improvement afforded to them than by monetary advantages during training. There is a great call for endowed scholarships of this nature and we hope to see many new ones instituted before another year has passed.

---

AN unusually complete three years' course is offered to women at the Royal College of Saint Katharine, Poplar, in preparation for the duties of health visitors and sanitary inspectors. The students reside at St. Katharine's, where the College forms part of the Medical Officer of Health's Welfare Scheme for the Borough of Poplar, having an ante-natal clinic, two dental, and six infant clinics. During the first two years the students take training at the Household and Social Science Department of King's College for Women, and the last year is devoted to training in children's and maternity hospitals, six months at each hospital. Lectures are also given on the religious basis of social work. Nurses who take this course are excused some part of it in consideration of their previous experience. It will be a misfortune if the new regulations for Health Visitors' training have the effect of curtailing this admirable course. Time will surely show that the requisite subjects for obtaining the new diploma cannot be properly mastered by ordinary students in less than three years.

---

AN important innovation is announced in respect to the holidays of matrons and nurses in the service of the Department of Soldiers' Civil Re-establishment. For the future they are to have two weeks holiday every six months. The ordinary Civil Service holiday allowance is eighteen days in the year, but this was quite obviously insufficient for members of the nursing staff. The institution of a six-monthly holiday instead of a yearly one has much to recommend it and would be popular in many establishments, although people who dislike any dislocation of routine would probably fear its effects in unsettling the staff and making extra work for everyone.

---

THE London County Council has taken a good lead in holding classes for the wives of Canadian soldiers awaiting their turn for embarkation. The marriages total some 1,000 a month and the new brides are by no means perfectly equipped for the conditions of life awaiting them on the other side. At the London County Council Women's Institute, Exmouth Street, Hampstead Road, N.W. 1, admirable courses are being given to prepare the emigrants for the tasks which await them in the way of cooking, laundry work, house-planning, lighting and heating, poultry-keeping, dairy work, etc. It is of the utmost importance that some elementary instruction in home nursing and monthly nursing should be added to these opportunities. If only one out of twenty of the intending emigrants could be assisted to take a full course of midwifery

**ROUND THE HOSPITALS**—(*continued*).

an enormous boon would be conferred on the Canadian settlers. If this be impracticable, at the least some effort should be made in every case to impart the elementary facts of asepsis and indicate the main dangers to avoid in childbirth, so that the mothers may be able to help each other.

Bermondsey Board of Guardians have decided to cancel all the agreements entered into with their medical and nursing staffs that in computing their superannuation allowances, the war bonuses shall not be taken into account. This vexed question was referred to in the columns of The Hospital on July 12, when it was then pointed out how unjustly these agreements worked. In the special case that brought this injustice prominently forward, the officer lost over £12 a year in the superannuation allowance, a serious item when spread over a number of years, but the guardians have decided to allow this amount, subject to the approval of the Ministry of Health. There is every possibility that the same agreement has been entered into by other boards of guardians with their staffs, and it will only be when a concrete case comes forward, as in the case of Bermondsey, where there is a serious loss in the superannuation allowances, that action will be taken to cancel the agreements, and justice will be done to the officers. There is little doubt that if these war bonuses had not been granted, there would have been substantial increases in the salaries.

The Central Midwives Board for Scotland held simultaneous examinations on July 28 and 29 at Edinburgh, Glasgow, and Dundee. At the Edinburgh centre there were 23 successful candidates, at Glasgow 35, and at Dundee 9, a total of 67.

At the Dudley Road Poor Law Infirmary, Birmingham, which has now been evacuated by the military authorities, the Infirmary Committee have granted a month's leave of absence to every member of the nursing staff in the employ of the Guardians, with board allowance of £2, in addition to £1 included in salary. The nurses are to be retained in the service of the Guardians at such work as the matron may think advisable until the infirmary is again in working order. There are many kindred institutions which would gladly receive members of the staff for temporary duty during the process of clearing up.

The importance of settling on a good name when regulating a matter of public health is illustrated in the decision at the Annual Conference of Urban District Councils of England and Wales to give to the Chairman of the Urban District Councils the title of Warden. A hope was expressed that the necessary Bill to give it sanction would be introduced into Parliament and carried into law. It is easy to understand that the word "Warden" thus secured will lend fresh dignity to the office and stimulate public recognition of its value to the community. In the same way the term "Nursing Sister," once secured by law to adequately trained members of the nursing services, would add fresh lustre to the profession, and command that due respect which is now too often ignorantly bestowed on impostors.

An appalling account of the ravages of influenza on the Labrador coast has been contributed by the Rev. W. W. Perrett, head of the Moravian Mission in that district, to the *Times*. The disease was brought to Labrador by a sailor on board the mission steamer about the middle of October, and ravaged the inhabitants like a forest fire. Within a short time house after house was filled with dead. There was no question of relief or nursing aid; the imperative call was for volunteers to bury the dead. At Hebron, the northernmost village, Bishop Martin and his wife by heroic efforts, saved seventy of the people; but the coast of Labrador is left desolate, as many of the inhabitants, men and women of fine physique, have perished. At Okak there are only 59 survivors out of 266, and at Nain and Hopedale, where smallpox and measles were added to the scourge, the survivors are still fewer. At the sealing-stations whole families died with none near to help.

